# In the Acute Phase of *Trypanosoma cruzi* Infection, Liver Lymphoid and Myeloid Cells Display an Ambiguous Phenotype Combining Pro- and Anti-Inflammatory Markers

**DOI:** 10.3389/fimmu.2022.868574

**Published:** 2022-05-26

**Authors:** Carina de Lima Pereira dos Santos, Natalia Vacani-Martins, Cynthia Machado Cascabulho, Mirian Claudia de Souza Pereira, Ian Nicholas Crispe, Andrea Henriques-Pons

**Affiliations:** ^1^ Laboratório de Inovaçõeses em Terapias, Ensino e Bioprodutos, Fundação Oswaldo Cruz, Instituto Oswaldo Cruz, Rio de Janeiro, Brazil; ^2^ Laboratório de Ultraestrutura Celular, Fundação Oswaldo Cruz, Instituto Oswaldo Cruz, Rio de Janeiro, Brazil; ^3^ Laboratory Medicine and Pathology, University of Washington Medical Center, Seattle, WA, United States

**Keywords:** hepatic immune response, *Trypanosoma cruzi* infection, immunological tolerance, liver, inflammation

## Abstract

Multiple cell populations, cellular biochemical pathways, and the autonomic nervous system contribute to maintaining the immunological tolerance in the liver. This tolerance is coherent because the organ is exposed to high levels of bacterial pathogen-associated molecular pattern (PAMP) molecules from the intestinal microbiota, such as lipopolysaccharide endotoxin (LPS). In the case of *Trypanosoma cruzi* infection, although there is a dramatic acute immune response in the liver, we observed intrahepatic cell populations combining pro- and anti-inflammatory markers. There was loss of fully mature Kupffer cells and an increase in other myeloid cells, which are likely to include monocytes. Among dendritic cells (DCs), the cDC1 population expanded relative to the others, and these cells lost both some macrophage markers (F4/80) and immunosuppressive cytokines (IL-10, TGF-β1). In parallel, a massive T cell response occured with loss of naïve cells and increase in several post-activation subsets. However, these activated T cells expressed both markers programmed cell death protein (PD-1) and cytokines consistent with immunosuppressive function (IL-10, TGF-β1). NK and NK-T cells broadly followed the pattern of T cell activation, while TCR-γδ cells appeared to be bystanders. While no data were obtained concerning IL-2, several cell populations also synthesized IFN-γ and TNF-α, which has been linked to host defense but also to tissue injury. It therefore appears that *T. cruzi* exerts control over liver immunity, causing T cell activation *via* cDC1 but subverting multiple populations of T cells into immunosuppressive pathways. In this way, *T. cruzi* engages a mechanism of hepatic T cell tolerance that is familiar from liver allograft tolerance, in which activation and proliferation are followed by T cell inactivation.

## Introduction

Chagas disease affects 6 to 8 million people in the Americas. However, it is no longer restricted to the continent, as chronically infected patients were identified in Japan, Europe, and Australia due to migratory movements (1). Since 1911, when the first pathological study of a deceased patient was performed [reviewed in (2)], a direct correlation was noted between the level of hepatic fatty degeneration and the morbidity of acutely infected patients. Microscopically, the authors observed, “…hypertrophy and hemosiderotic pigmentation in Kupffer cells, with a small lymphomonocytic infiltrate inside dilated intralobular or periportal capillaries” (2). Although the hepatic pathology was described more than a century ago, liver involvement in the morbidity of infected patients was overlooked for decades. However, liver pathogenesis has received more attention since the observation that acute oral infection caused a more severe liver involvement (3).

Today we know that the liver is important in the clearance of blood trypomastigote forms (4) and that the disease carries additional risk in patients with non-alcoholic steatohepatitis (NASH), as it exacerbates hepatic injury (5). Accordingly, acutely infected patients have very high levels of hepatic transaminases and activated C protein, with lower levels of coagulation factor VII (6). Although the infection of hepatocytes is not high as in the case of macrophages or cardiomyocytes *in vivo*, these results indicate the profound impact of the infection on this cell type.

The liver contains an abundant resident macrophage population, the Kupffer cells (KCs), many of which are long-lived and derived from the yolk sac or the fetal liver (7), but these are supplemented with blood monocyte-derived cells particularly after injury, inflammation or infection (8). Liver myeloid cells also include classical dendritic cells (cDCs), mostly the cDC2 subset (9). Both KCs and liver cDCs may promote T cell tolerance, rather than full activation, and this likely contributes to the phenomenon of liver tolerance (10). Liver T cell tolerance is manifest not as direct inactivation, but as sub-optimal activation that leads rapidly to a tolerant or immunosuppressive state, and which may feature death of the responding T cells (11, 12). The liver also contains abundant NK cells and NK-T cells, and a minor population of TCR-γδ cells (γδT lymphocytes).

Few published data describe the phenotype and function of liver cell subpopulations after *T. cruzi* infection. NK cells increased up to six-fold after seven days of infection and were the primary source IFN-γ (13), a protective cytokine for the infection (14). Hepatic NKT cells are divided into type I, or invariant NKT (iNKT), with semi-invariant T cell receptors (TCR) that recognize glycolipid antigens, and type II NKT cells. Type II cells express more diverse TCRs and recognize microbial phospholipids and sulfatides, besides glycolipids. Both NKT cell types recognize antigens in the context of CD1d (15), and it was observed that *T. cruzi* infected CD1d^-/-^ mice, which lack type I and II NKT cells, have a milder infection with reduced liver mononuclear cell infiltration (16). On the other hand, mice that lack only iNKT cells have a more severe infection, with higher mortality rates. The authors suggested that iNKT cells dampen the inflammatory response, possibly regulating type II NKT cells that would be pro-inflammatory (16).

Only splenic, bone marrow-derived, and monocyte-derived DCs were studied in the context of *T. cruzi* infection, and most results suggest that the parasite suppresses their antigen presenting function (17, 18), especially in susceptible mouse strains (19, 20). In most cases, there was a reduction in the expression of MHC-II and co-stimulatory molecules, reduced endocytic capacity, and increased production of anti-inflammatory cytokines and PD-L1 after host DC infection or interaction with parasite molecules. *T cruzi* infection results in hepatomegaly and liver inflammation, consistent with a strong immune response; however the parasite is not effectively cleared in chronic patients. This is comprehensible in terms of liver tolerance, a mechanism of organ-specific T cell inactivation that was first document in the context of allograft transplantation (21–24).

To determine whether the hepatic immune response to *T. cruzi* was consistent with such liver tolerance, we evaluated the response of all of these cell types during infection with *T cruzi*.

## Materials and Methods

### Mice

All experiments were performed using eight-week-old specific pathogen-free (spf) male C57BL/6 mice obtained from the Universidade de Campinas (CEMIB). All mice were housed for at least one week before experimentation under conditions complying with the “Guide for the Care and Use of Laboratory Animals” (DHEW Publication No. NIH 80- 23, 1996). The FIOCRUZ Committee of Ethics in Research approved this project (L006/15 and L-020/2019-A1), according to resolution 196/96 of the National Health Council of the Brazilian Ministry of Health.

### *Trypanosoma cruzi* Infection

For experimental infection, bloodstream trypomastigote forms of *T. cruzi* Y strain were obtained from infected Swiss-Webster mice at seven days post-infection (dpi) (25). The parasites were counted, and the intraperitoneal (IP) inoculum was adjusted in PBS to 1×10^4^ parasites per mouse in 100 µL. Control mice received 100 µL of PBS.

### Isolation of Liver Cells

Immediately after euthanasia, the livers were perfused *via* the portal vein with 15mL of DMEM (Gibco, WA, Massachusetts, USA) plus CaCl2 5mM (Sigma-Aldrich, St. Louis, MO, USA) at 37°C. The organs were gently removed, the Glisson capsule was cut two or three times, and perfused with another 30mL of digestion medium (DMEM plus CaCl2 5mM and 30 units of collagenase type I (Thermo Fisher Scientific) and 30 units of collagenase type II (Worthington, Columbus, OH, USA). The isolated cells were washed (237 x *g* for 10 minutes at 4°C), passed through a 40µm strainer (Greiner Bio-One, Kremsmuenster, Austria), and kept in ice-cold DMEM supplemented with 10% of FCS (Gibco).

### Percoll-Based Separation of Debris

After dissociation and centrifugation, pelleted cells were resuspended in 10 mL of ice-cold DMEM supplemented with 10% FCS and carefully placed over 30mL of Percoll Plus (Gibco) (diluted in PBS to 20%). The samples were centrifuged at 420 x *g* for 30 minutes at 20°C and pelleted cells were resuspended in 1 mL of red blood cell lysis solution (hypotonic PBS diluted 1:10 in distilled water) for 13 seconds and immediately washed in PBS 1x.

### Flow Cytometry – Multiparametric Analysis

For flow cytometry analysis, the primary tissue-isolated cells were incubated for 30 minutes in ice-cold DMEM supplemented with 10% FCS and 10% inactivated sheep serum to block Fcγ receptors. Viable, phase-bright cells were counted using a Neubauer chamber and maintained on ice for antibody labeling. The cellular concentration per well in U-bottomed 96-wells plates was adjusted for 3×10^5^ intrahepatic and 1×10^6^ splenic cells. The samples were then incubated for 30 minutes with previously titrated anti-mouse monoclonal antibodies (mAbs) against surface markers, washed twice using ice-cold DMEM, and permeabilized using the FoxP3 Fixation/Permeabilization Buffer (Biolegend, San Diego, CA, USA), following the manufacturer’s protocol. Intracellular cytokines were labeled with mAbs for 30 minutes, the cells were washed twice, and the acquisition was made using a CytoflexS (Beckman Coulter, Brea, CA, USA) flow cytometer at the Multiparametric Multiuser Flow Cytometry Facility at the Instituto Oswaldo Cruz. Cellular viability was evaluated using a solution of 7-Amino-Actinomycin D 10% (7-AAD) (BD Biosciences, Franklin Lakes, NJ, USA), and data analysis was carried out using CytExpert (version 2.1) software. CD3 and CD11c labeling was used to define a wide gate that included the lymphoid and myeloid cells analyzed (**Supplementary Figure 1**). The doublet exclusion was performed using FSC-H x FSC-A dot plots, and the gating strategy used for each analysis is shown in the Figures.

**Figure 1:**
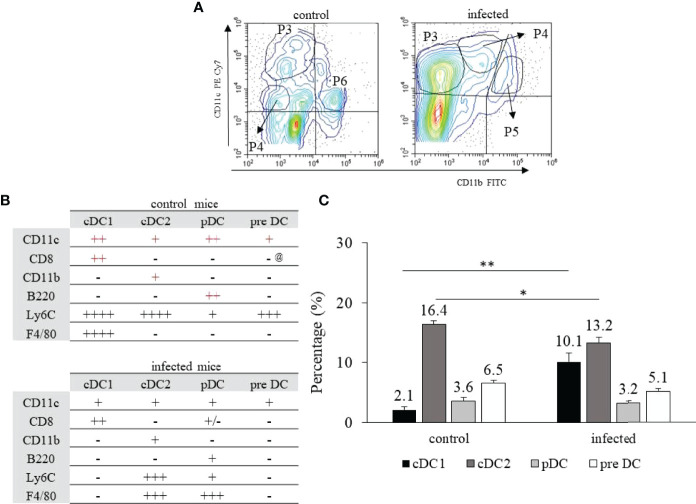
Hepatic dendritic cells’ identification and cellular frequency after *T. cruzi* infection. C57BL/6 mice were infected with 1x10^4^ blood trypomastigote forms of *T. cruzi* Y strain and on dpi 15, infected and control mice were euthanized. The HDCs we isolated by enzymatic dissociation and primarily identified according to the expression of CD11c and/or CD11b **(A)**. In control mice, cDC1 and pDC were identified in P3, cDC2 in P6, and pre DC in P4. In infected mice, cDC1 and pre DC were identified in P3, pDC in P4, and cDC2 in P5 **(A)**. The complete phenotype to discern cDC1, cDC2, pDCs, and pre DCs is depicted in B and the frequency of each cellular population is shown in **(C)** @ Indicates that most pre DCs from control mice do not express CD8 and, therefore, were represented as (-), but about 15% of the cells were CD8^+^. The primary canonic phenotype for each population is shown in red **(B)**. * means statistical significance (p ≤ 0.05) using the Kruskal Wallis test followed by Dunn’s post-test, and ** means p ≤ 0.01 using the one-way ANOVA test followed by the Tukey’s post-test.

The functional identification of naïve and antigen-primed T lymphocytes was defined as follows: naïve T lymphocytes CD62L^+^CD44^low^; effector CD62L^-^CD44^high^CD127^-^, effector memory (EM) CD62L^-^CD44^high^CD127^+^; and central memory (CM) CD62L^+^CD44^high^CD127^+^. The identification of hepatic dendritic cells (HDCs) was based on the expression of CD11c, CD11b, CD8, B220, F4/80, and Ly6c, and the subpopulations discerned were cDC1 (lymphoid DC); cDC2 (myeloid DC); pDC, and pre DC. The gating strategy for HDCs identification is shown in **Figure 1B** and individual markers for control mice are shown in **Supplementary Figure 1**, and for *T. cruzi* infected mice on dpi 15 are shown in **Supplementary Figure 2**. The identification of KCs was based on F4/80 labeling, and the gating strategy is shown in **Figure 3**. Three independent experiments were made to study intrahepatic cell populations, with seven mice per group.

List of mAbs used (all from BD): F4/80 PE Texas red (clone T45-2342), CD11b FITC (clone M1/70), CD11c PE CY7 (clone HL3), LY-6C APC CY7 (clone AL-21), CD8 PerCP (clone 53-6.7), SIGLEC H APC (clone 440c), MHC-I BV421 (clone M1/42), MHC-II Alexa 488 (clone M5/114), PDL-1 PE (clone MIH5), CD3 ALEXA 700 (2C.11), CD4 PERCP (clone RM4-5), CD44 PE (clone IM7), CD62L APC CY7 (clone MEL-14), CD127 PECY7 (clone EBIOSB/199), PD-1 APC (clone J43), CD152 (CTLA- 4) PE CF594 (clone UC10-4F10-11), CD3 PE (clone 2C.11), NK1.1 PECY7 (clone PK136), TCR γδ APC (clone GL3), PD-1 APC Cy7 (clone J43), TGF-β1 PerCP Cy 5.5 (clone TW7-16B4), TGF- beta BV421 (clone TW7-16B4), TNF-α BV510 (clone MP6-XT22), IFN-γ BV650 (clone XMG1.2, IL-10 BV605 (clone JES5-16E3).

### Statistical Analysis

All data are expressed as arithmetic mean ± SD. First, we used the Shapiro–Wilk test (RStudio, Boston, MA, USA; URL http://www.rstudio.com/) to identify what data groups had a Gaussian or a nonparametric distribution. Then, we used GraphPad Prism version 7.05 to apply the one-way ANOVA test followed by Tukey’s post-test for Gaussian distributions. For nonparametric data, we used Kruskal Wallis and Dunn’s post-test. The results were considered significant when the *p*-value was ≤ 0.05.

## Results

Our first goal was defining the phenotypic and possible functional variations of HDCs after *T. cruzi* infection, once this population exerts immunoregulatory functions that can dictate the resulting immune response in the organ. We observed four subpopulations in control and infected mice, primarily identified in CD11c x CD11b dot plots (**Figure 1A**), with the complete phenotypes depicted in **Supplementary Figures 1**, **2**, and **Figure 1B**. The populations were cDC1 (in the P3 gate for control and infected mice); cDC2 (in P6 for control and P5 for infected mice); pDC (in P3 for control and P4 for infected mice); and pre DC (in P5 for control and P4 for infected mice) (**Figure 1A**). After infection, there was a 5-fold increase in cDC1 HDCs (**Figure 1C**), with a discrete but significant reduction in the frequency of cDC2 cells (**Figure 1C**). HDCs are strategic professional APCs capable of modulating T lymphocytes’ function. Considering splenic cDC1 cells, they participate in blood pathogen clearance, in the uptake of dead blood cells, and, depending on the context, antigen uptake can lead to cross-tolerance or cross-priming (26). Accordingly, BATF3-deficient mice that lack cDC1 cells have impaired virus-specific cytotoxic T lymphocyte (CTL) responses and ineffective tumor rejection (27). Classical DC1 cells also express multiple Toll-like receptors (TLR) (28), including receptors that were described in sensing *T. gondii* (29), leading to IL-12 secretion that contributed to early antiparasite defense (30). In *T. cruzi* infection, however, primarily splenic and bone-marrow-derived DCs were studied. It was described that they down-modulate the endocytic capacity, the expression of co-stimulatory and MHC molecules, and upregulate the expression of IL-10, TGF-β1, IL-4, and PD-L1 [reviewed in (18)]. Moreover, it was observed that the expression of IL-12, IL-6, TNF-α, HLA-DR, and CD40 by monocytes-derived DCs was drastically reduced after infection (31).

**Figure 2:**
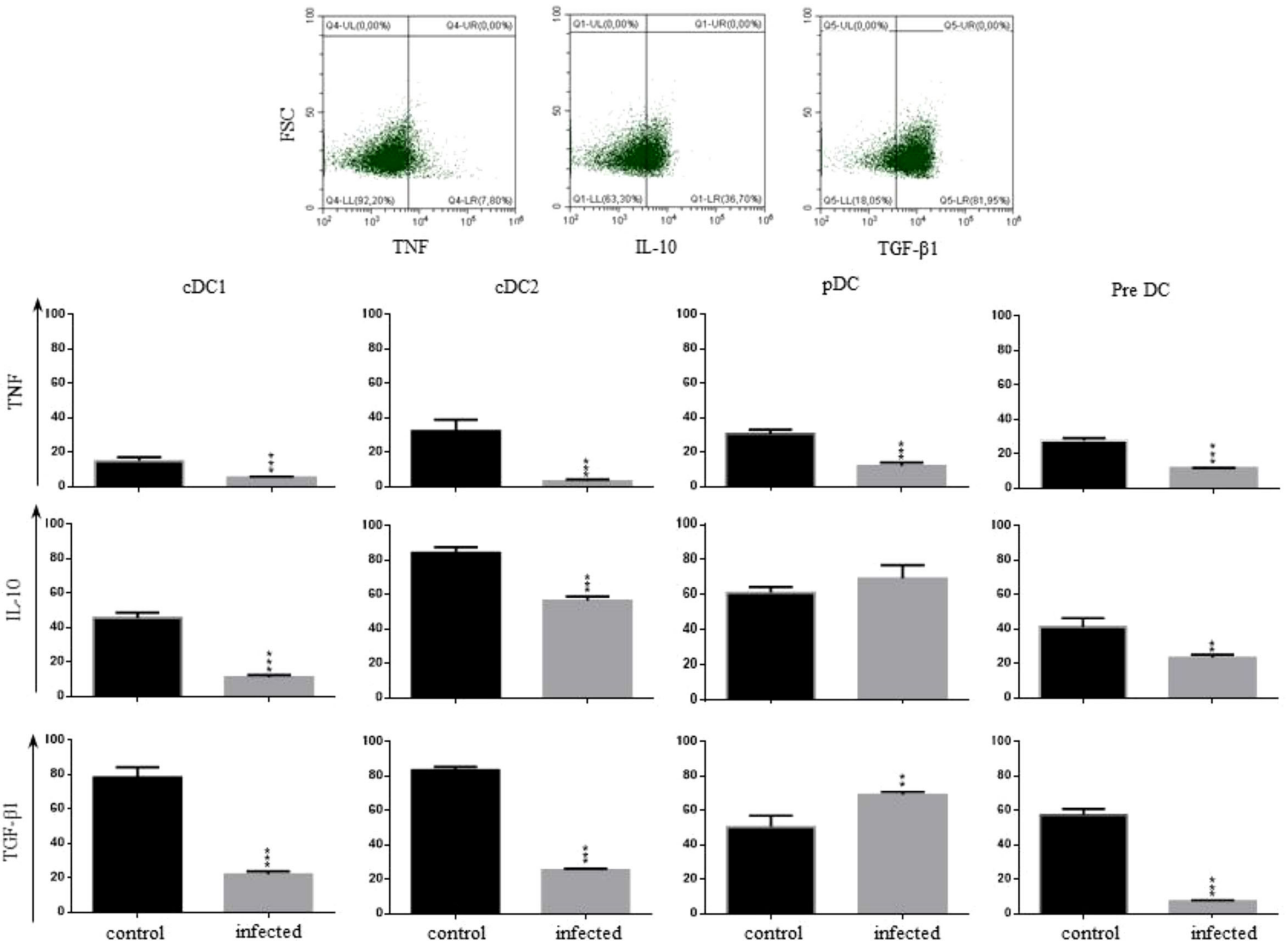
Profile of cytokines produced by hepatic dendritic cells: The definition of TNF-α^+^, IL-10^+^, or TGF-β1^+^ cells was done as illustrated in the dot plots. The frequency of cDC1, cDC2, pDCs, and pre DC producing TNF-α, IL-10, or TGF-β1 was evaluated by flow cytometry in control and *T. cruzi* infected mice on dpi 15, with the complete gating strategy shown in **Supplementary Figures 1** (control) and **2** (infected mice). Statistically significant differences are shown as ** p ≤ 0.05 and ***p ≤ 0.01 using the one-way ANOVA test followed by Tukey’s post-test.

In addition to evaluating the frequency of HDC subpopulations, it is essential to assess the modulation of anti-inflammatory cytokines produced by these cells after infection. We then evaluated the production of IL-10 and TGF-β1, besides TNF-α (pro-inflammatory), by all HDCs identified in control and *T. cruzi* infected mice (**Figure 2**). Due to the high levels of PAMPs from the bacterial flora that HDCs are continuously exposed to, these cells secrete mainly tolerogenic cytokines under considered steady-state conditions (32). Therefore, cDC1, cDC2, pDC, and pre DC from the liver of control mice produced relevant levels of mostly IL-10 and TGF-β1 (**Figure 2**). However, this profile changed after the *T. cruzi* infection. Regarding cDC1, the frequency of cells producing IL-10 and/or TGF-β1 reduced significantly, although with a negligible percentage of cells producing TNF-α after infection (**Figure 2**). Moreover, they mainly were MHC-I^+^ and/or MHC-II^+^ (**Supplementary Figure 3**) and B7^+^ cells (data not shown), while less than 15% of the cDC1 cells expressed PD-L1 (**Supplementary Figure 3**). Therefore, this population could play a role in activating Th1-biased T lymphocytes, being protective cells in the balance between acute tolerance versus inflammation in the liver. On the other hand, the cDC2 population was ambiguous regarding its role in the inflammatory response after infection. Although the infection led to a reduced frequency of IL-10^+^ cells, up to 60% of the events still produced this cytokine. Likewise, there was a reduction in the frequency of TGF-β1^+^ cells after infection but, in this case, no more than 20% of the events were positive (**Figure 2**). Classical DC2 cells were mostly MHC-I and -II DP cells before and after infection, with virtually no cells expressing PD-L1 (**Supplementary Figure 3**). On the other hand, pDCs reinforced their role as down-modulatory cells, with up to 75% of the cells producing IL-10 and an increased percentage of cells producing TGF-β1 after infection (**Figure 2**). Up to 70% of the cells were MHC-I and -II DP cells, and no more than 10% of the cells were PD-L1^+^ (**Supplementary Figure 3**).

Regarding pre DCs, they are Siglec-H^-^ and Ly6C^-^ when committing into the cDC1 lineage, while pre DC that differentiate into cDC2 are Siglec-H^-^ and Ly6C^+^ (33). We observed that about 70% of the cells from control mice were CD8^-^Ly6C^+^ (**Supplementary Figure 1**), a phenotype compatible with pre cDC2 cells. However, an average of 15% of the pre DCs were CD8^+^, suggesting that they were pre cDC1 cells (**Supplementary Figure 1**). Among HDCs from control mice, pre DCs composed the population with the lowest frequency of cells expressing MHC-II (as MHC-I and -II DP cells) (**Supplementary Figure 3**) and also with the lowest level of expression per cell (data not shown). Less than 5% of the cells were MHC-II SP cells (data not shown), and about 15% on average were MHC-I SP cells (**Supplementary Figure 3**). After the infection, we observed no other markers besides CD11c, suggesting a commitment into pre cDC1 cells (33). Moreover, there was a 3-fold increase in MHC-I SP cells, no alteration in the frequency of cells expressing MHC-II, and a decrease in PD-L1^+^ cells (**Supplementary Figure 3**). Confirming their commitment to the phenotype of pre cDC1 cells after infection, their profile of cytokines was similar to cDC1 cells (**Figure 2**). Therefore, despite the subtle modulations in the frequency of cDC2, pDCs, and pre DCs after infection in the liver, the *in vivo* infection with *T.cruzi* functionally affected all HDC populations.

**Figure 3:**
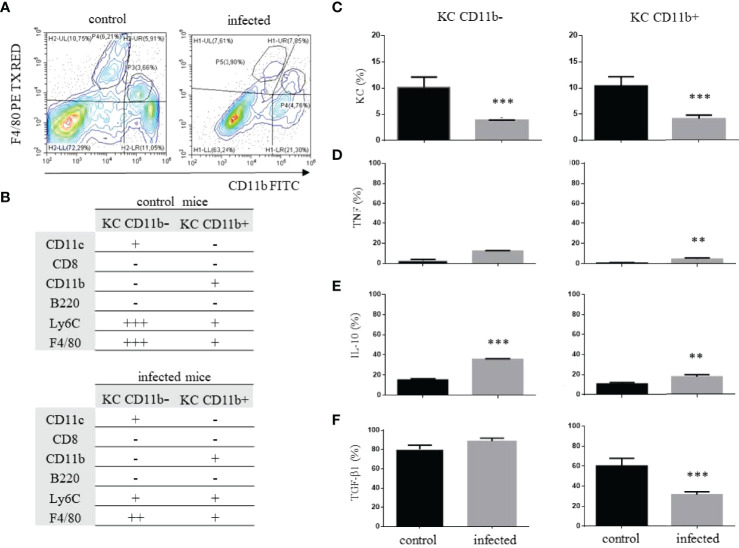
Kupffer cell identification and phenotype after *T. cruzi* infection. C57BL/6 mice were infected with trypomastigote forms of *T. cruzi* Y strain and on dpi 15, infected and control mice were euthanized. The KCs were identified as F4/80^+^ and two subpopulations were depicted according to the expression of CD11b **(A)**. The complete phenotype to discern CD11b^-^ and CD11b^+^ KCs is shown in **(B)** and the frequency of each cellular population is shown in **(C)**. The frequency of each subpopulation expressing TNF-α **(D)**, IL-10 **(E)**, or TGF-β1 **(F)** are indicated for control and infected mice. ** means statistical significance (p ≤ 0.05) and *** means p ≤ 0.01. We used the Kruskal Wallis test followed by Dunn’s post-test to analyze TNF-α and TGF-β1 in CD11b- KC cells. We used the one-way ANOVA test followed by Tukey’s post-test for all other analyses.

We evaluated the phenotypic changes imposed by the infection on KC, one of the liver’s main APCs and scavenger cell populations. In our hands, two F4/80^+^ populations could be discerned based on the expression of CD11b, which were F4/80^+^CD11b^-^ and F4/80^+^CD11b^+^ (**Figure 3A**), and their extended phenotype is shown in **Figure 3B**. An average of 10% of both subpopulations were found in control mice, and the infection led to a reduction, with less than 5% of cells after infection (**Figure 3C**). The frequency of CD11b^-^ KCs producing TNF-α, even after infection, was very low (**Figure 3D**), while the frequency of cells producing IL-10 increased after infection (**Figure 3E**). Moreover, about 80% of these cells produced TGF-β1 either in control or infected mice (**Figure 3F**). Most CD11b^-^ KCs expressed MHC-I and about 40% expressed MHC-II (**Supplementary Figures 4A, B**), with an increase of more than 3-fold of CD11b^-^ KC expressing PD-L1 after infection (**Supplementary Figure 4C**). Therefore, these cells seem to perform downregulatory biological functions in the liver, unlike CD11b^+^ KCs that seem to be pro-inflammatory. In this case, the frequency of cells producing TNF-α slightly increased, and IL-10 or TGF-β1 were not the prevalent cytokines produced after infection (**Figures 3D, F**). Most cells expressed MHC-I and -II, and less than 5% expressed PD-L1 (**Supplementary Figures 4A, C**).

**Figure 4:**
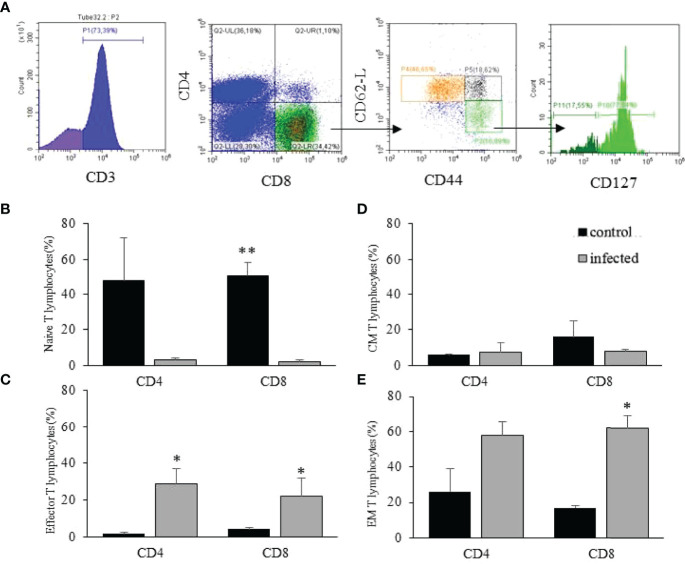
Intrahepatic T lymphocytes after *T. cruzi* infection. The flow cytometry data analysis was done for CD4^+^ and CD8^+^ T lymphocytes (illustrated in A) in the gate of CD3^+^ events. The gating strategy is shown for EM and effector cells using an infected mouse **(A)**. Then, for each population, the frequency of naïve (CD62L^+^CD44^low^); effector (CD62L^-^CD44^high^CD127^-^), EM (CD62L^-^CD44^high^CD127^+^); and CM T lymphocytes (CD62L^+^CD44^high^) was analyzed **(A)**. The distribution of naïve **(B)**, effector **(C)**, CM **(D)**, and EM **(E)** T lymphocytes in the liver of control and infected mice on dpi 15 are shown. * means p ≤ 0.05 and ** means p ≤ 0.005 using the one-way ANOVA test followed by Tukey’s post-test.

We then evaluated the phenotypic alterations imposed by the *in vivo* infection over CD4^+^ and CD8^+^ intrahepatic T lymphocytes. For the analysis, we divided the cells into naïve, effector, EM, and CM T lymphocytes (**Figure 4A**). In control mice, up to 70% of the CD4^+^ or CD8^+^ T lymphocytes were naïve (**Figure 4B**), and less than 5% of the events in our wide analysis gate (**Supplementary Figure 1**) were identified as effector T lymphocytes (**Figure 4C**). There was an inversion after the infection, and we observed that näive intrahepatic CD4^+^ and CD8^+^ T lymphocytes reduced drastically to less than 5% (**Figure 4B**). Conversely, intrahepatic CD4^+^ and CD8^+^ effector T lymphocytes increased more than 5-fold after infection (**Figure 4C**). Regarding CM T lymphocytes, there were no significant alterations in this population after infection for CD4^+^ and CD8^+^ T cells (**Figure 4D**). On the other hand, CD8^+^ EM T lymphocytes increased more than 5-fold after infection, but there was no statistically significant difference for CD4^+^ EM T lymphocytes (**Figure 4E**).

**Figure 5:**
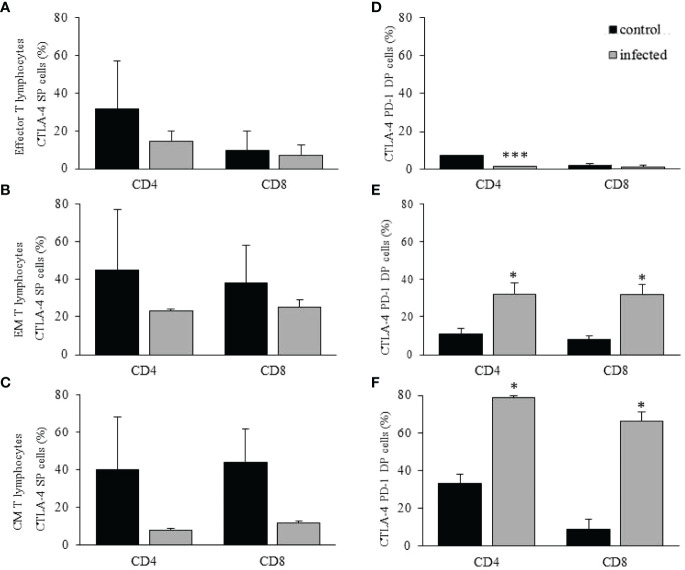
Expression of immunoregulatory molecules on intrahepatic T lymphocytes. The analysis of CTLA-4^+^ single positive **(A–C)** or CTLA-4^+^PD-1^+^ double-positive (DP) **(D–F)** cells is shown for CD4^+^ or CD8^+^ T intrahepatic lymphocytes. The cell frequency was evaluated in control and infected mice on dpi 15. * means p ≤ 0.05 and *** means p ≤ 0.001 using the one-way ANOVA test followed by Tukey’s post-test.

Although the primary phenotype that identifies antigen-primed T lymphocytes indicated that the infection led to an augmented frequency of effector (CD4^+^ and CD8^+^) and EM (CD8^+^) T lymphocytes, it is necessary to evaluate the expression of immunomodulatory molecules and the profile of cytokines produced. We then analyzed the frequency of PD-1 and/or CTLA-4 positive cells, two main immunomodulatory molecules that downregulate T lymphocytes**’** function in the liver (34). Using the same gating strategy shown in **Figure 4**, we observed no differences when comparing control with infected mice regarding the CTLA-4 SP phenotype in effector (**Figure 5A**), EM (**Figure 5B**), or CM (**Figure 5C**) CD4^+^ or CD8^+^ T lymphocytes. There were no more than 20% of the cells as CTLA-4 SP after infection in all populations (**Figures 5A–C**). Regarding the CTLA-4^+^PD-1^+^ DP phenotype, there were less than 5% of effector CD4^+^ or CD8^+^ T lymphocytes in the infected group (**Figure 5D**), the most expanded population after infection (**Figure 4C**). Although up to this point, intrahepatic effector T lymphocytes seem to be functional non-tolerogenic cells after *in vivo* infection, the profile of cytokines secreted is required for further conclusions.

Regarding EM (**Figure 5E**) and CM (**Figure 5F**) T lymphocytes as CTLA-4^+^PD-1^+^ DP cells, there was a significant increase in both populations after infection, a phenotype compatible with tolerogenic functions. There were less than 5% of PD-1 SP cells in any group or T lymphocyte population (data not shown).

**Figure 6:**
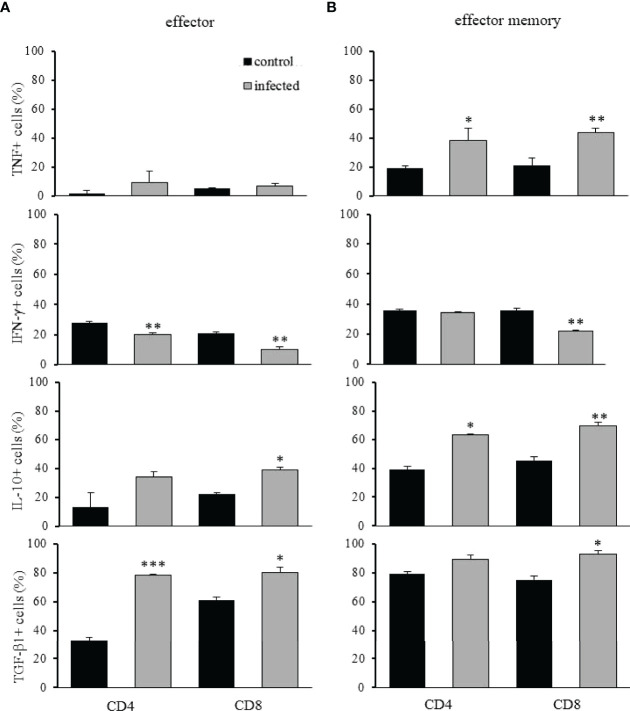
Pro- and anti-inflammatory cytokines produced by effector and effector memory T lymphocytes after *T. cruzi* infection. The analysis of TNF-α, IFN-γ, IL-10, and TGF-β1 positive CD4^+^ or CD8^+^ intrahepatic T lymphocytes are shown as indicated. The cellular frequency of effector and effector memory T cells was evaluated in control and infected mice on dpi 15. * means p ≤ 0.05, ** means p ≤ 0.005, and *** means p ≤ 0.001 using the one-way ANOVA test followed by the Tukey’s post-test.

To further infer the cellular function of intrahepatic T lymphocyte subpopulations after infection, we evaluated the production of anti- and pro-inflammatory cytokines. We observed that, although expanded effector T lymphocytes were mostly CTLA-4 and PD-1 negative cells, up to 80% secreted TGF-β1 and up to 40% produced IL-10 (**Figure 6A**). Less than 15% of effector CD4**^+^** or CD8**^+^** T lymphocytes produced TNF-α (**Figure 6A**) and no more than 30% produced IFN-γ. Our results suggest that these cells may have a balanced function in the infection, do not clearly indicating a pro-inflammatory role. Moreover, 60% to 90% of CD4**^+^** or CD8**^+^** EM T lymphocytes produced IL-10 and/or TGF-β1 (**Figure 6B**), with up to 40% of the cells producing TNF-α and IFN-γ, and no clear indication of immunological function after infection. Probably, these populations were heterogeneous, and further phenotypic analyses would better discern subpopulations with pro or anti-inflammatory functions. Yet, the maintenance of tolerogenic pathways seems to be the primary outcome for antigen-primed intrahepatic T lymphocytes after infection.

**Figure 7:**
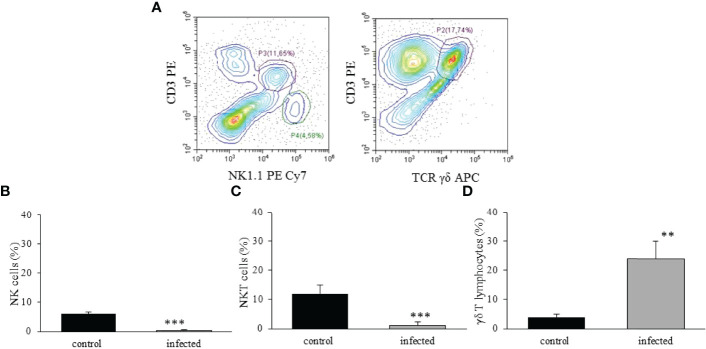
Analysis of NK, NKT, and γδ T lymphocytes in the liver of *T. cruzi* infected mice. C57BL/6 mice were IP infected with *T. cruzi*, and the hepatic cells were isolated by enzymatic dissociation on dpi 15. The identification of NK (NK1.1^+^CD3^-^), NKT (NK1.1^+^CD3^+^), and γδ T lymphocytes (CD3^+^γδ TCR^+^) are shown in **(A)** For better visualization of the populations, a dot plot from a control mouse illustrates the analysis gate of NK and NKT cells, and a dot plot from an infected animal shows the gate of γδ T lymphocytes. The frequency of each cell population is indicated **(B–D)** in control and infected mice. ** means p ≤ 0.05 and *** means p ≤ 0.01. For NK and γδ T lymphocytes, we used the one-way ANOVA test followed by the Tukey’s post-test, and for NKT cells we used the Kruskal Wallis followed by the Dunn’s post-test.

Considering NK (NK1.1^+^CD3^-^), NKT (NK1.1^+^CD3^+^), and γδ T lymphocytes (CD3^+^ γδ TCR^+^) in the liver (**Figure 7A**), we observed a significant reduction of NK (**Figure 7B**) and NKT (**Figure 7C**) cells after infection. On the other hand, γδ T lymphocytes increased about 5-fold on dpi 15 (**Figure 7D**).

**Figure 8:**
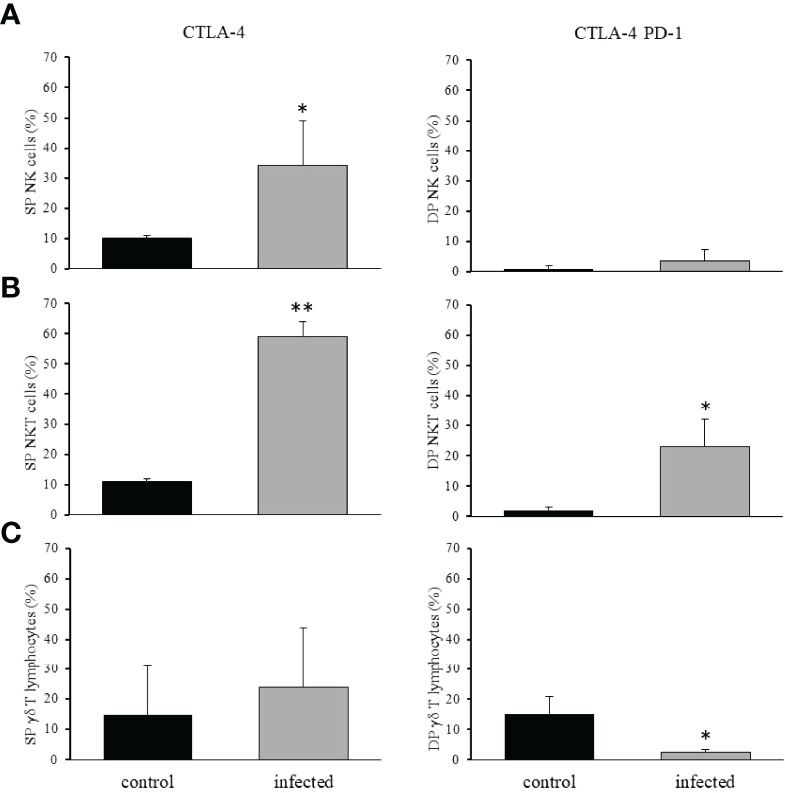
Expression of immunomodulatory molecules by NK, NKT, and γδ T lymphocytes. The intrahepatic cells were isolated from control and *T. cruzi* infected mice on dpi 15, and NK **(A)**, NKT **(B)**, and γδ T lymphocytes **(C)** were analyzed. CTLA-4 single positive cells (left panels) or PD-1 and CTLA-4 double-positive cells (right panels) are shown. * means p ≤ 0.05 and ** means p ≤ 0.005. We used the one-way ANOVA test for NK and NKT cells followed by the Tukey’s post-test, and for γδ T lymphocytes, we used the Kruskal Wallis test followed by the Dunn’s post-test.

It has already been published that the interaction of PD-1 with its ligand PD-L1 downregulates NK cell function and leads to anergy (35). Similarly CTLA-4 has also been described as a down regulatory molecule for NK cells (36). After *T. cruzi* infection, we observed an increase in the frequency of hepatic NK cells as CTLA-4 SP, and no alteration in the frequency of NK cells with the CTLA-4^+^PD-1^+^ DP phenotype (**Figure 8A**). Both molecules have also been described as downregulatory components for NKT cells (37, 38), and we observed a significant increase in CTLA-4^+^PD-1^+^ DP and CTLA-4^+^ SP NKT cells after infection (**Figure 8B**). Finally, γδ T lymphocytes are also under the surveillance of both immunoregulatory molecules (39, 40), and although γδ T lymphocytes rarely express CTLA-4, they can upregulate PD-1 upon activation. After infection, we observed no alteration in the frequency of cells as CTLA-4^+^ SP, and there was a reduction in CTLA-4^+^PD-1^+^ DP γδ T lymphocytes (**Figure 8C**).

**Figure 9:**
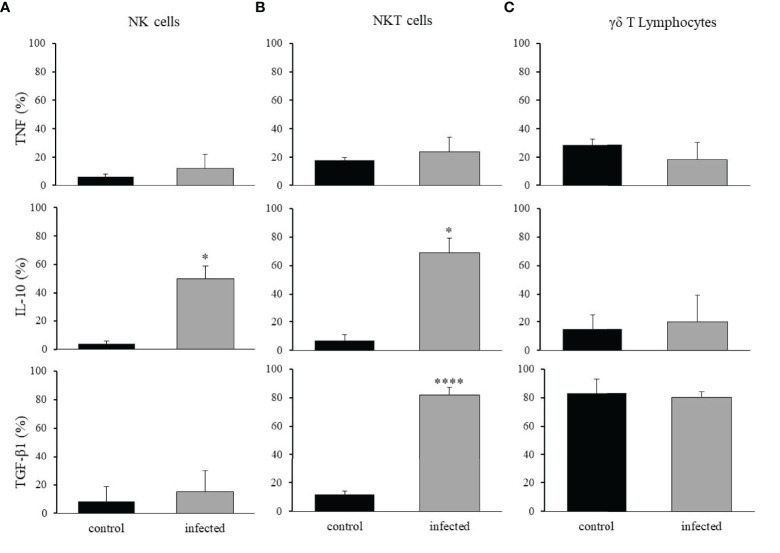
Production of anti- and pro-inflammatory cytokines by NK, NKT, and γδ T lymphocytes. The intrahepatic cells were isolated from control and *T. cruzi* infected mice on dpi 15, and the production of TNF-α, IL-10, or TGF-β1 was analyzed in NK **(A)**, NKT **(B)**, and γδ T lymphocytes **(C)**. * means p ≤ 0.05, **** means p ≤ 0.001 using the Kruskal Wallis test followed by the Dunn’s post-test.

**Figure 10:**
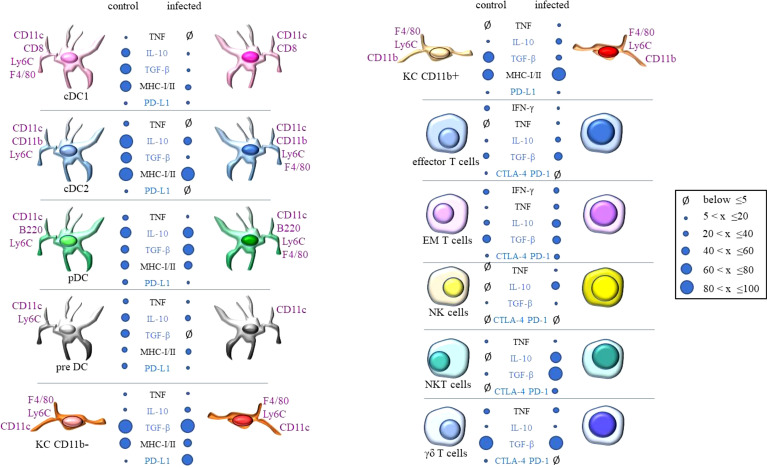
Summary of phenotypic modulations observed in hepatic cells from control and *T. cruzi* infected mice obtained on dpi 15 (acute phase). The results obtained by flow cytometry were stratified in this chart according to the frequency of cells expressing each marker. Subpopulations with up to 5% of frequency were considered not represented. The dimension of each dot proportionally represents the frequency of each subpopulation found in flow cytometry analysis. According to the legend, the intervals vary from 5% to 20% of positive cells, 20% (exclusive) to 40%, and so on. Effector and EM lymphocytes represent the results for CD4 or CD8 cells.

When considering the cytokines produced by each cell population, we observed in control mice that less than 20% of the NK or NKT cells produced TNF-α, IL-10, or TGF-β1 (**Figures 9A, B**). However, after infection, the frequency of NK cells expressing IL-10 increased about 6-fold (**Figure 9A**), and the frequency of NKT cells expressing IL-10 and TGF-β1 increased 13-fold and 8-fold, respectively (**Figure 9B**). Regarding the expanded population of γδ T lymphocytes, we observed no significant alteration in any of the cytokines studied after the infection, but about 80% of these cells produced TGF-β1 (**Figure 9C**). Therefore, although the NK and NKT cell populations were reduced substantially after infection, they assumed anti-inflammatory immune functions. This was similar to γδ T lymphocytes that, despite very few cells expressing CTLA-4 or PD-1, mostly continued to produce TGF-β1. A summary of all phenotypic changes identified is shown in **Figure 10**.

## Discussion

Ancient infection by *T. cruzi* was identified in mummies dating back nine thousand years (41), and Chagas disease was described more than a century ago. However, it still poses a challenge to Public Health management for most Latin American countries. It is also a challenge for researchers who study the pathophysiological mechanisms involved in the different clinical manifestations of the disease, mainly cardiac or digestive. Chagasic chronic cardiomyopathy, the leading cause of death, is usually observed decades after the acute phase and manifests in 30 to 35% of patients. Therefore, it is natural that cellular populations that compose cardiac inflammatory foci and contribute to cardiac pathogeny are studied in much more detail (42, 43) than intrahepatic cell populations. However, the oral infection leads to a usually aggressive acute phase that includes liver impairment, and it is known that a more severe acute phase is associated with a higher morbidity and mortality in chronic patients (44). This led us to focus on immune cells in the liver in Chagas disease.

It is long known that the *T. cruzi* infection leads to a robust inflammatory response in the periphery, with polyclonal activation of B and T lymphocytes (45) and a protective Th1-biased response (46, 47). Still, the parasite persists in chronic patients (48), as evidenced by recurrence of infection after cardiac transplantation from *T. cruzi* infected donors, in the case of HIV/AIDS and *T. cruzi* coinfection, and under pharmacological immunosuppression (49). Therefore, immune escape mechanisms probably coexist with immunological protective pathways, resulting in cardiomyopathy in only subset of the chronically infected patients. We speculate that hepatic tolerogenic cells could participate in the prevention of Chagasic cardiomyopathy for most chronically infected Chagas patients. This proposed function for hepatic cells is based on our observation that the liver is the only compartment where tolerogenic cells and pathways were found during the infection. In agreement with our proposed function for tolerogenic hepatic cells, previous work has been published using bone marrow-derived DCs induced into a tolerogenic phenotype by *in vitro* culture with dexamethasone. These tolerant DCs were adoptively transferred into *T. cruzi* infected mice and it was observed that they controlled chronic cardiac inflammation and fibrosis (50). Although the authors did not consider hepatic cells, we can extend their observation and propose that tolerogenic hepatic cells would continuously exit the liver. Then, these cells would balance the inflammatory extra-hepatic environment and be important in suppressing parasite-induced cardiomyopathy. When these liver-dependent check and balance mechanisms are inefficient, the systemic immune response would favor cardiac pathogenesis, as observed in up to 35% of the patients.

If systemic and hepatic pro- and anti-inflammatory pathways contend for the generation of parasite-induced Chagasic cardiomyopathy, a prerequisite would be the exit of hepatic cells from the liver with tolerogenic properties against cognate antigens. This possibility was raised decades ago for other pathologies but still lacks conclusive scientific confirmation. For example, it was observed that a previous liver transplant increases the rate of engraftment in the case of a second organ transplanted from the same donor (51), with hepatic donor-derived leukocytes reaching central lymphoid organs within two hours (52). Moreover, this hepatic tolerogenicity theory can be illustrated by the induction of oral and portal venous tolerance, since the administration of antigens by the oral route or by the portal intravenous route induces both local and systemic tolerance (53, 54). Another possibility is that recirculating immune cells acquire tolerogenic properties while transiting through the liver. This possibility was confirmed by the prior administration of donor splenic or bone marrow-derived cells *via* the portal vein, which promoted peripheral tolerance to posterior skin grafts from the same donor (55, 56). This induced tolerance can be reversed by a portocaval bypass, avoiding the cellular passage through the liver, which confirms the role of the organ in inducing oral tolerance (57).

Finally, the expression of exogenous proteins in the liver through transgenesis induced specific systemic tolerance to those proteins. This approach leads to Treg cells induction, which suppresses antibody production and the CD8^+^ T lymphocytes response (58). The most studied exogenous gene used was factor IX to treat severe hemophilia type B (59).

While we hypothesize that the exit of liver cells with tolerogenic functions could contribute to the protection against chronic cardiomyopathy, we have recently published the other side of the coin (60). In this case, we observed that peripheral T lymphocytes induced a partial shift towards a pro-inflammatory response in the liver after infection. Unfortunately, few data are available describing the importance of hepatic cells in *T. cruzi* infection, and much remains to be clarified about the interplay between intrahepatic and peripheral cells in the pathogenesis or control of *T. cruzi* infection. However, the available data using non-hepatic cells reinforces the tolerogenicity hypothesis, as the liver is constitutively rich in anti-inflammatory mediators, especially IL-10 and TGF-β1. For example, non-hepatic DCs exposed to autocrine IL-10 have a reduced capacity to activate allogeneic T cells and prime naive T cells into a Th1 profile in the presence of LPS (61) or *Mycobacterium* sp. (62).

In the present work, we show that the *in vivo* infection with *T. cruzi* triggers intrahepatic cells into ambiguous phenotypes, with subpopulations of hepatic DCs, KCs, and lymphoid populations that express both tolerogenic and inflammatory markers. Regarding liver DCs, cDC1 cells exhibited a phenotype compatible with inflammatory functions, and the pre DCs seem to be committed into cDC1 cells after infection. On the other hand, pDCs and, at a lower extent, cDC2 cells seem to have tolerogenic functions. Similarly, KCs can be divided into CD11b^-^ cells, most of which produced tolerogenic mediators, and CD11b^+^ cells that seemed more inflammatory. Even CD4^+^ and CD8^+^ effector T lymphocytes, effector memory T cells, and NK cells have a significant proportion of cells secreting IL-10 and/or TGF-β1, counterbalancing cells that were TNF-α^+^ and IFN- γ^+^. NK cells and γδ T lymphocytes were predominantly tolerogenic after infection, and while we did not evaluate parenchymal cells, LSECs, or HSCs on the grounds that they are not expected to leave the organ and exert immunomodulatory functions in the periphery, we acknowledge these cells might also influence T cell tolerance locally. Additional functional experiments are necessary to test the possibility of tolerogenic hepatic cells controlling the development of chronic cardiomyopathy.

Therefore, we can conclude that the numerous biochemical pathways that maintain the liver as a tolerogenic organ under steady-state conditions, remain active even in the presence of circulating *T. cruzi* parasites during acute infection. This hepatic tolerogenic status is also maintained in other pathogenic conditions, leading to persistent infection by viruses and other protozoa. Although this characteristic allows a reduced regimen of immunosuppressants to manage liver transplanted patients, it is becoming more apparent that the unconventional pathways that govern the hepatic immune response must be studied in a systematic way. Only this way, the whole picture of cells and biological pathways that compose the active network of the immune system will reveal potential therapeutic targets for numerous diseases beyond the *T. cruzi* infection.

## Data Availability Statement

The original contributions presented in the study are included in the article/**Supplementary Material**. Further inquiries can be directed to the corresponding author.

## Ethics Statement

The animal study was reviewed and approved by The FIOCRUZ Committee of Ethics in Research approved this project (L006/15 and L-020/2019-A1), according to resolution 196/96 of the National Health Council of the Brazilian Ministry of Health.

## Author Contributions

CLPS: executed and analyzed all experiments, manuscript review. NVM and CMC: helped in the execution of experiments. IC and MSP: participated in the construction of the rationale, manuscript review and editing. AH-P: supervised the work, participated in the construction of the rationale, wrote the manuscript. All authors have read and agreed to the submitted version of the manuscript.

## Funding

The authors would like to thank the Fundação Carlos Chagas Filho de Amparo à Pesquisa do Estado do Rio de Janeiro (FAPERJ) grant number E-26/010.002422/2019 and Fundação Oswaldo Cruz, Instituto Oswaldo Cruz.

## Conflict of Interest

The authors declare that the research was conducted in the absence of any commercial or financial relationships that could be construed as a potential conflict of interest.

## Publisher’s Note

All claims expressed in this article are solely those of the authors and do not necessarily represent those of their affiliated organizations, or those of the publisher, the editors and the reviewers. Any product that may be evaluated in this article, or claim that may be made by its manufacturer, is not guaranteed or endorsed by the publisher.
