# Supplementary figures and images for: In the Acute Phase of Trypanosoma cruzi Infection, Liver Lymphoid and Myeloid Cells Display an Ambiguous Phenotype Combining Pro- and Anti-Inflammatory Markers
Source: Front Immunol. 2022 May 26;13:868574. doi: 10.3389/fimmu.2022.868574 (PMC9204308; doi:10.3389/fimmu.2022.868574)

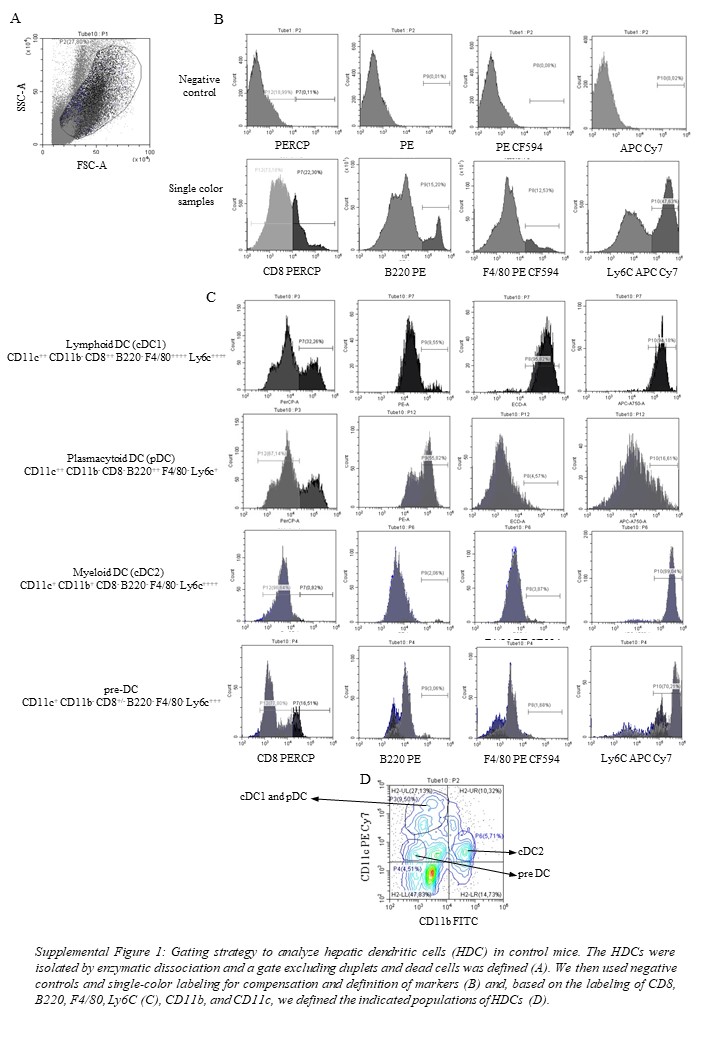

Supplement: Supplementary file 1 [file Image_1.jpeg]

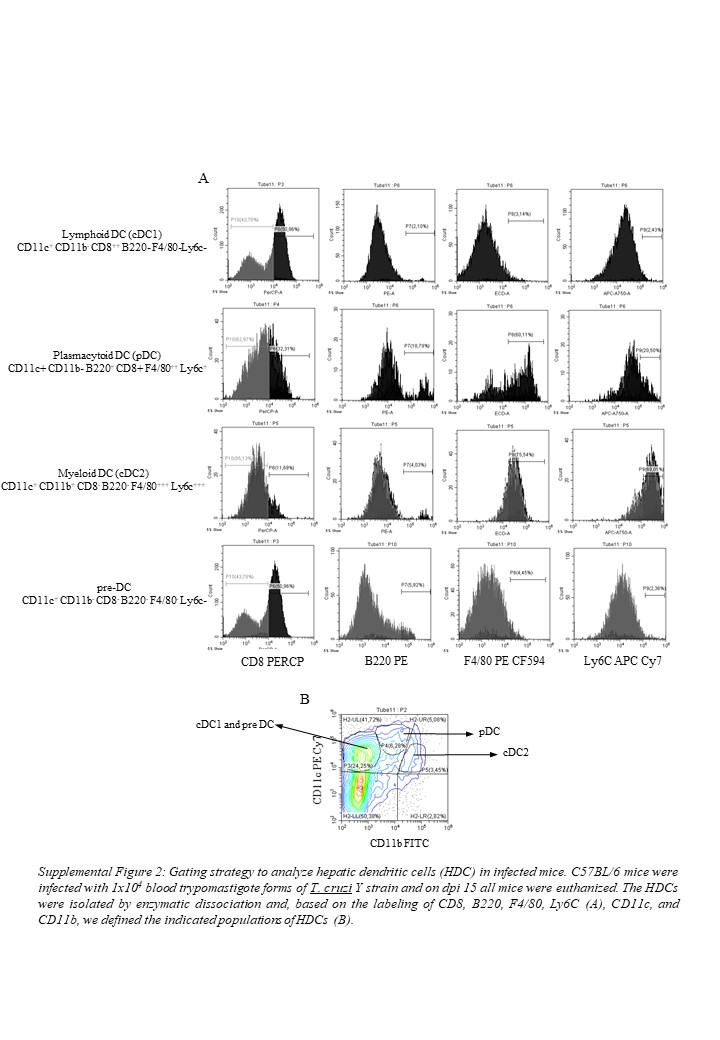

Supplement: Supplementary file 2 [file Image_2.jpeg]

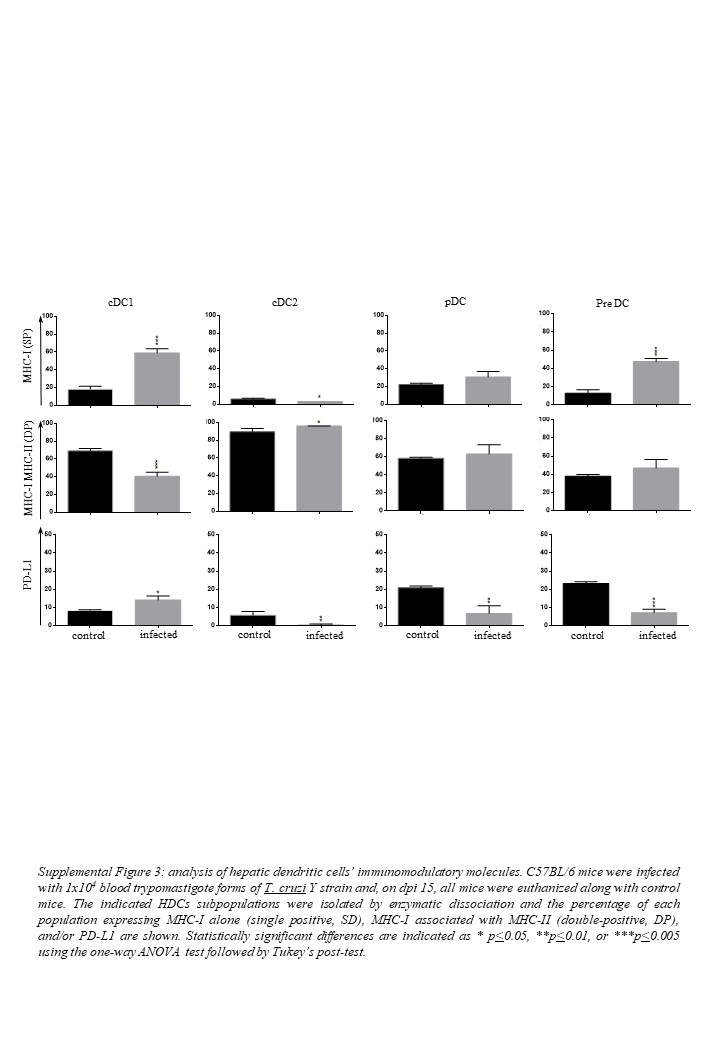

Supplement: Supplementary file 3 [file Image_3.jpeg]

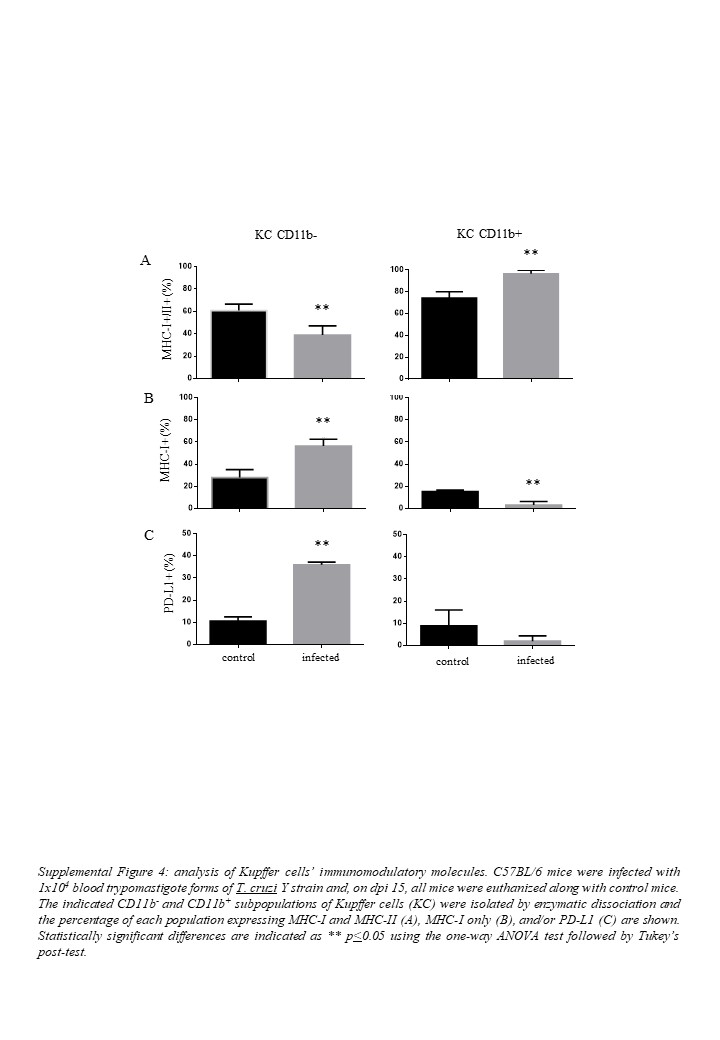

Supplement: Supplementary file 4 [file Image_4.jpeg]
